# Tuning of oxygen vacancy-induced electrical conductivity in Ti-doped hematite films and its impact on photoelectrochemical water splitting

**DOI:** 10.1038/s41598-020-64231-w

**Published:** 2020-05-04

**Authors:** Pranab Biswas, Ardak Ainabayev, Ainur Zhussupbekova, Feljin Jose, Robert O’Connor, Aitkazy Kaisha, Brian Walls, Igor V. Shvets

**Affiliations:** 1 School of Physics and Centre for Research on Adaptive Nanostructures and Nanodevices (CRANN), Trinity College Dublin, Dublin 2, Ireland; 2 School of Physical Sciences, Dublin City University, Glasnevin, Dublin 9, Ireland

**Keywords:** Energy science and technology, Materials science

## Abstract

Titanium (Ti)-doped hematite (α-Fe_2_O_3_) films were grown in oxygen-depleted condition by using the spray pyrolysis technique. The impact of post-deposition annealing in oxygen-rich condition on both the conductivity and water splitting efficiency was investigated. The X-ray diffraction pattern revealed that the films are of rhombohedral α-Fe_2_O_3_ structure and dominantly directed along (012). The as-grown films were found to be highly conductive with electrons as the majority charge carriers (n-type), a carrier concentration of 1.09×10^20^ cm^−3^, and a resistivity of 5.9×10^−2^ Ω-cm. The conductivity of the films were reduced upon post-deposition annealing. The origin of the conductivity was attributed firstly to Ti^4+^ substituting Fe^3+^ and secondly to the ionized oxygen vacancies (V_O_) in the crystal lattice of hematite. Upon annealing the samples in oxygen-rich condition, V_O_ slowly depleted and the conductivity reduced. The photocurrent of the as-grown samples was found to be 3.4 mA/cm^−2^ at 1.23 V vs. RHE. The solar-to-hydrogen efficiency for the as-grown sample was calculated to be 4.18% at 1.23 V vs. RHE. The photocurrents were found to be significantly stable in aqueous environment. A linear relationship between conductivity and water-splitting efficiency was established.

## Introduction

Typical resources to meet the world’s progressive energy-needs, such as coal and petroleum are limited and have been largely depleted. In addition, the burning of fossil fuels is causing global warming and climate change by emitting carbon dioxide into the atmosphere, which is a major threat to human civilization^1^. As a result, interest in alternative, non-conventional, non-polluting, and renewable fuels has rapidly increased in an unprecedented way across the world, which can ensure a sustainable development. In this context, hydrogen (H_2_) can be considered as one of the most promising green fuels^2^. Amongst existing technologies, hydrogen evolution by photocatalytic dissociation of ubiquitous water in a photoelectrochemical cell (PEC) is one of the simple processes to convert solar energy into a storable chemical energy in the form of H_2_^3^.

A PEC essentially needs to have a photocatalytic semiconducting electrode that can absorb sunlight and induce the electrochemical reactions in the cell by producing excess electrons in the conduction band^3^. This leads to splitting of water into oxygen and hydrogen even below the typical dissociating voltage of 1.23 V^4^. Notably, hematite (α-Fe_2_O_3_) has attracted much attention in recent times as a promising photocatalytic material^5–7^. Being abundant in nature, nontoxic, stable in aqueous environments, and suitable to absorb visible-range electromagnetic spectra, hematite emerges as one of the most competitive candidates for photoanode. With a band gap of around 2.2 eV, hematite can achieve a maximum solar-to-hydrogen (STH) conversion efficiency of around 15% theoretically, which stands well above the STH threshold efficiency of 10% required for practical applications^8–10^. Other conventional materials for water splitting, such as TiO_2_ and WO_3_ have a maximum theoretical STH conversion efficiency of 1% and 6%, respectively^11^. For hematite, the existing reports show that the maximum efficiency is still far below from both the threshold and theoretical maximum STH efficiencies^3,4,10^. It was found that its efficacy is partially hindered by a low electrical conductivity and sluggish reaction kinetics. These can be improved by using high quality hematite films and by increasing carrier concentration in the films. It was reported that water-splitting properties of hematite could be improved by inducing oxygen vacancies in its lattice^12–15^. However, these studies do not provide much insight on the conductivity dependent efficiency.

In previous reports, researchers have synthesized α-Fe_2_O_3_ by using several techniques, such as atomic layer deposition (ALD)^16^, atmospheric pressure chemical vapour deposition (APCVD)^17^, DC magnetron sputtering^18^, which are significantly expensive. Magnan *et al*.^19^ synthesized Ti-doped α-Fe_2_O_3_ by using molecular beam epitaxy (MBE) and tuned the doping concentration up to 17%. However, STH conversion efficiency was not reported for these films. In some papers , low-cost methods, such as hydrothermal^20,21^ and spray pyrolysis^22,23^ have also been reported. Apart from being cost-effective and relatively low-temperature (100–500 °C) method, spray pyrolysis-deposited films are reproducible and tuneable as well. Moreover, it leads to relatively high-quality and uniform films unlike hydrothermal methods. The above studies indicate the potential of hematite as a promising photoanode. These studies mainly focus on the enhancement of sunlight absorption by means of nanostructures and doping, while lacking insights on electrical conductivity and its impact on water splitting. They have obtained an average photocurrent density of around 0.5 to 2 mA/cm^2^ at 1.23 V vs. RHE (reversible hydrogen electrode) corresponding to a STH conversion efficiency of around 0.5 to 1.5%.

In this report, we have comprehensively studied combined effect of Ti-doping and oxygen vacancies on conductivity of hematite films deposited by using spray pyrolysis technique. We have tuned the oxygen vacancies in hematite by varying the annealing temperature in an oxygen-rich ambiance, while the deposition was carried out in an oxygen-depleted condition in a closed chamber. A relationship has been established between the water splitting efficiency and oxygen vacancy concentration.

## Results and discussion

**Figure 1:**
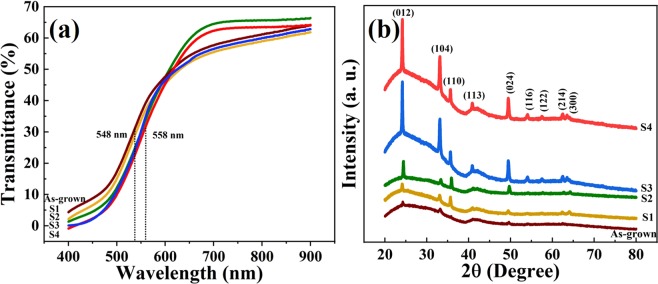
(**a**) Transmittance spectra of the as-grown, S1, S2, S3, and S4 samples indicating a sharp absorption around 550 nm corresponding a bandgap of 2.2 eV. (**b**) X-ray diffraction pattern showing peaks related to hematite films predominantly directed along (012).

The as-grown films, which were deposited in oxygen-poor condition were annealed in oxygen-rich condition for 30 min at different temperatures and denoted as S1 (100 °C), S2 (200 °C), S3 (300 °C), and S4 (400 °C), as summarized in Table 1. In order to study the optical properties of the samples, UV-Vis transmission spectroscopy was carried out. Figure 1(a) shows transmittance spectra of the samples in the electromagnetic spectrum range of 400 to 900 nm^24^. The sharp absorption at around 548 nm for the as-grown sample corresponds to an optical bandgap of 2.26 eV and at 558 nm for S4 sample corresponds to a bandgap of 2.22 eV. The reported bandgap of hematite (α–Fe_2_O_3_) was found to be 2.1-2.2 eV^25–28^. The small change in optical bandgap of 0.04 eV was attributed to relaxation in crystal-strain during post-deposition annealing in oxygen-rich ambiance. Figure 1(b) shows the X-ray diffraction (XRD) patterns of the films. The XRD peaks are in accordance with the characteristic peaks of crystalline rhombohedral structure of hematite, as recorded in JCPDS – International Centre for Diffraction Data No 33-0664^29,30^. The predominated growth-direction of the films was found to be along (012). It was observed that the peaks slightly shifted towards lower 2θ value (≈0.2 °), which was attributed to the strain in the hematite lattice structure due to Ti doping and its relaxation on annealing^31^. Figure SI 1 in the supplementary information (SI) shows the overlay of the peak depicting the shift on annealing.

**Table 1:** Name and description of the samples under investigation along with their resistivity values.

Sample Name →	As-grown	S1	S2	S3	S4
Description → (S1-S4 post-annealed for 30 min in air)	Without post-treatment	100 °C	200 °C	300 °C	400 °C
Resistivity (×10^−2^ Ω-cm) →	5.9	6.1	7.3	8.7	12.6

**Figure 2:**
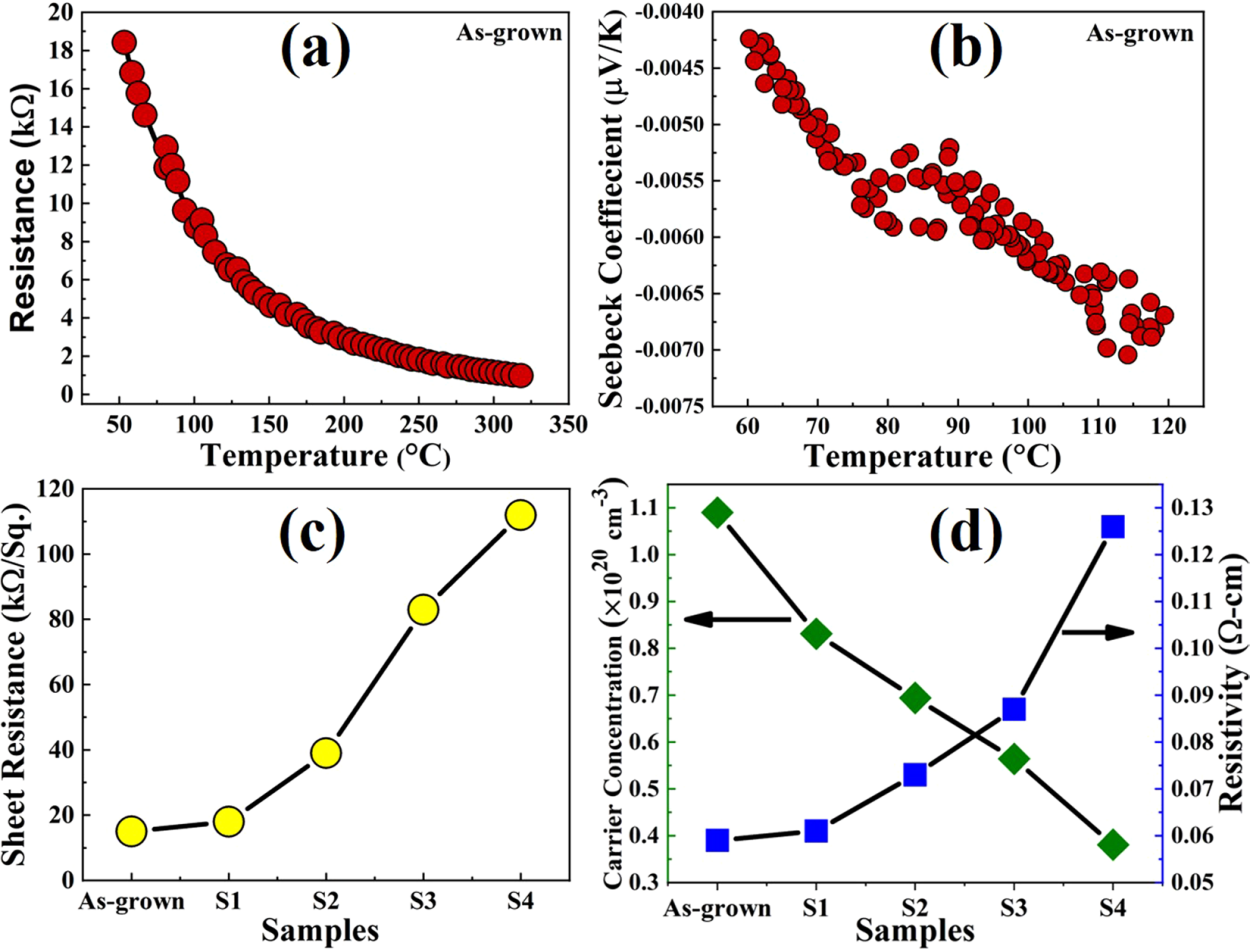
(**a**) Resistance as a function of temperature in vacuum of as-grown sample reveals typical semiconducting behaviour of the spray deposited hematite films. (**b**) Seebeck coefficient as a function of temperature. A negative coefficient indicates n-type conductivity of the films with electrons as the majority carrier and the corresponding values in the range of µV/K indicates a doped semiconductor with high carrier concentration. (**c**) Sheet resistance of all the samples reveals better conductivity of the as-grown sample and it gradually decreases upon annealing in oxygen-rich conditions. (**d**) Carrier concentration and resistivity of all the samples indicating a significantly conductive hematite films. Carrier concentration decreases and resistivity increases gradually on annealing the samples in oxygen-rich condition.

Figure 2(a) shows resistance of the as-grown sample as a function of temperature in the range of 53 to 318 °C. A negative slope of resistance with an increase in temperature indicates typical semiconducting behaviour of the hematite films. Figure 2(b) shows the Seebeck coefficients of the as-grown sample as a function of temperature in the temperature range of 60 to 120 °C. The Seebeck effect accounts for build-up of a potential difference (ΔV) across two ends of a semiconductor or conductor on applying temperature gradient, ΔT = T_hot_ – T_cold_ along the sample bar. The temperature gradient drives the majority charge carriers towards the cold side and produces a gradient in the number of carriers between the two ends leading to the potential difference. In case of a semiconductor, the majority of charge carrier determines both the sign of the ΔV and the Seebeck coefficient (α), which is defined as^32^,-

1 $$\alpha =-\frac{\Delta V}{\Delta T}$$

When it is positive (α > 0), then the semiconductor is said to be p-type, whereas semiconductors with electrons as the majority charge carrier give a negative value of Seebeck coefficient (α < 0). Most significantly, the magnitude of α indicates the carrier concentration and density of states in the semiconductor^32^. It was found that undoped semiconductors exhibit a Seebeck coefficient in the order of mV/K, whereas doped semiconductors show a value of the same in the range of µV/K. Upon applying a temperature from 60 °C up to 120 °C at the hot end of the sample, a Seebeck coefficient values was observed in the range of –0.0043 down to –0.0070 µV/K, respectively, as shown in Fig. 2(b). Therefore, the Ti-doped hematite films are n-type in nature and the value of Seebeck coefficient indicates a doped semiconductor with high carrier concentration. Figure 2(c) shows the sheet resistance of all the samples at room temperature. The sheet resistance for as-grown, S1, S2, S3, and S4 samples was calculated to be approximately 15, 18, 39, 83, and 112 kΩ/sq., respectively. The change in sheet resistance was attributed to the change in the density of oxygen vacancies upon annealing the samples in oxygen-rich condition. The carrier concentration and resistivity are shown in Fig. 2(d). The carrier concentration decreases with increased annealing temperature, whereas the resistivity increases on increasing the annealing temperature. The as-grown hematite film was found to have the highest carrier concentration of 1.09×10^20^ cm^−3^ and the lowest resistivity of 5.9×10^−2^ Ω-cm. On the other hand, sample S4 showed the lowest carrier concentration of 3.81×10^19^ cm^−3^ and a resistivity of 12.6×10^−2^ Ω-cm. Since the hematite films were deposited in an oxygen-depleted condition, more oxygen vacancies likely to be present in the as-grown sample, and hence, shows better conductivity, whereas post deposition annealing in an oxygen-rich environment gives rise to relatively resistive films due to introduction of oxygen in the lattice.

**Figure 3:**
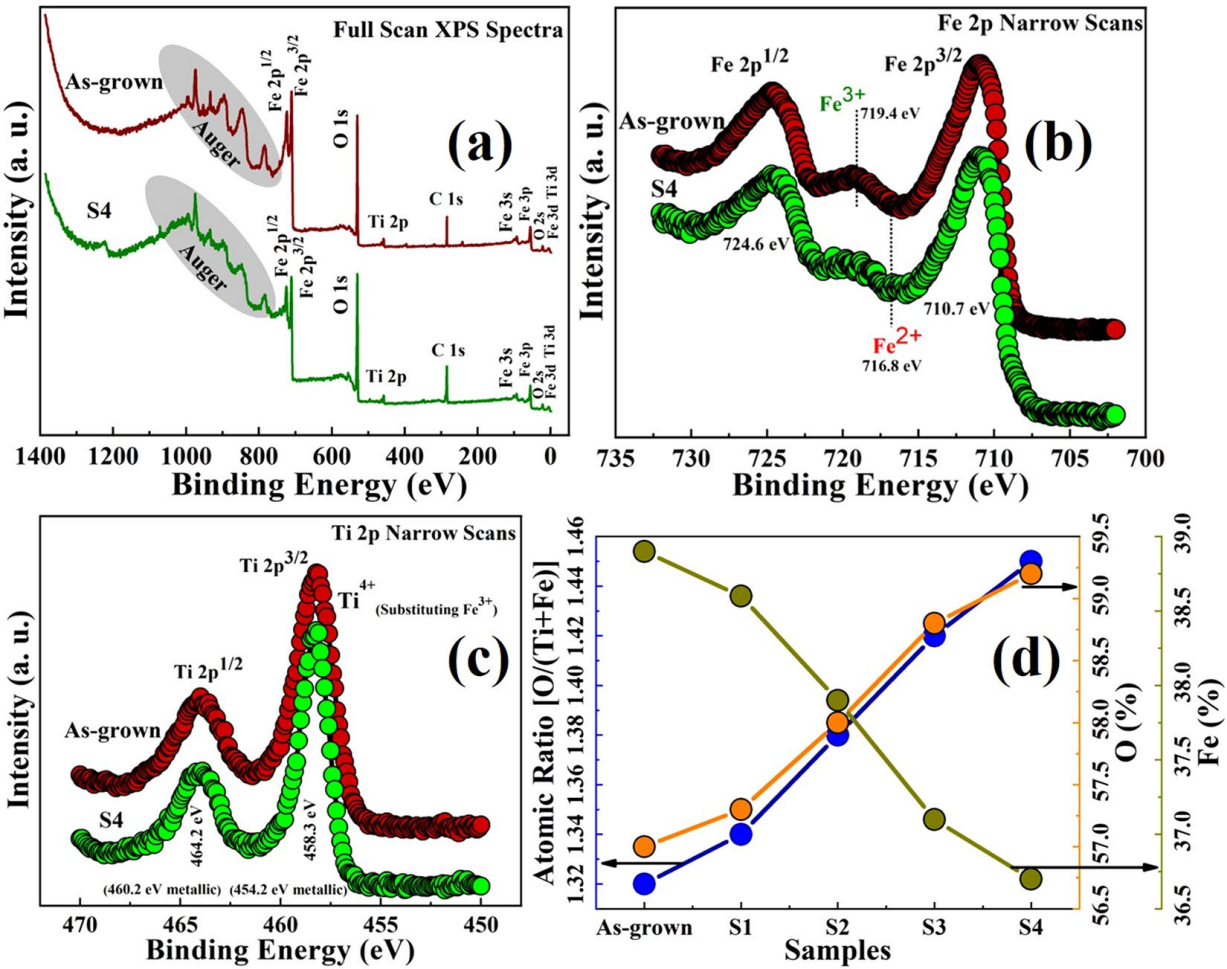
(**a**) Full scan XPS spectra of as-grown sample and S4 indicating the elemental composition and purity of the samples showing the peaks related to Ti, Fe, O, and C. (**b**) Fe 2p narrow scan XPS spectra of as-grown sample and S4, revealing an oxidation state of +3 of iron with a satellite peak at around 719.4 eV. (**c**) Ti 2p narrow scan XPS spectra of as-grown sample and S4 indicating +4 oxidation state of Ti substituting a Fe^3+^ ion in hematite lattice. It also indicates that metallic Ti is absent in the samples. (**d**) Oxygen and Fe Atomic percentage and O/(Ti+Fe) atomic ratio of all the samples (as-grown + S1 + S2 + S3 + S4) revealing the presence of oxygen vacancies maximum in the as-grown sample grown in the oxygen-depleted ambiance. The vacancies decrease with an increasing in annealing temperature in an oxygen-rich condition.

**Figure 4:**
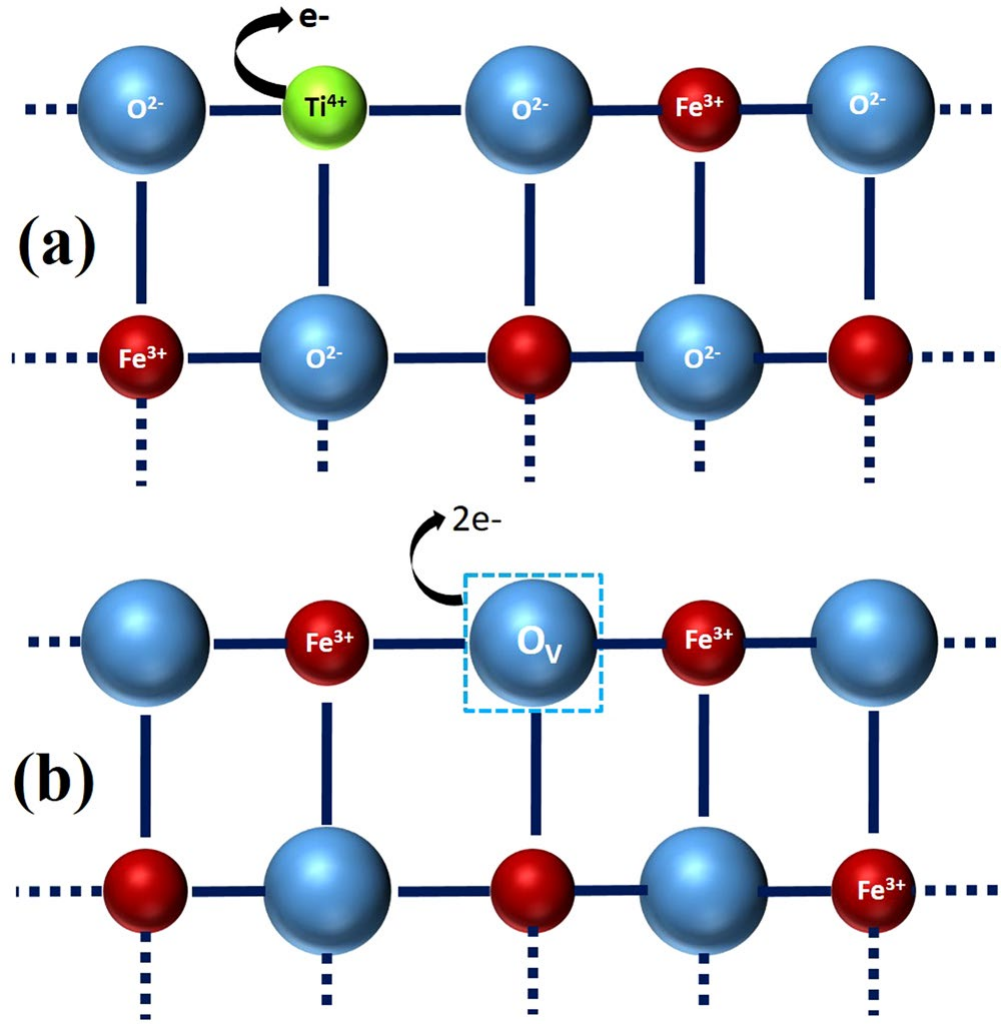
Schematic depicting a simplistic view of 2-dimensional lattice of Ti-doped hematite. (**a**) In Ti-doped α-Fe_2_O_3_, a Ti^4+^ ion substitutes a Fe^3+^ ion, donating charge carrier in the lattice. (**b**) An ionized oxygen vacancy in the hematite adding charge carriers to the lattice.

The origin of the conductivity in the hematite films was systematically investigated by using X-ray photoelectron spectroscopy (XPS). All the samples were sputtered for 15 minutes in order to get rid of surface contamination. The XPS data revealed that the samples contain no other element than Ti, Fe, and O, as shown in the full scan spectra of as-grown and S4 samples in Fig. 3(a). The full scans, Fe 2p narrow scans, and Ti 2p narrow scans XPS spectra of samples S1, S2, and S3 are presented as Fig. SI 2(a,b,c), respectively, in the supplementary information. The Fe2p narrow scan XPS spectra of as-grown and S4 samples are shown in Fig. 3(b) indicating two major peaks at 710.7 eV and 724.6 eV, corresponding to Fe 2p3/2 and Fe 2p1/2 with a spin orbital splitting (SOS) value of 14.1 eV. The typical satellite peak at 719.4 eV reveals that the films are of hematite with ‘+3’ oxidation state of iron^3^. Figure 3(c) shows Ti 2p narrow scan XPS spectra of as-grown and S4 samples with 2 major peaks at 458.3 eV and 464.2 eV corresponding to Ti 2p3/2 and Ti 2p1/2, respectively, separated by a SOS value of 5.9 eV. The electrons in Ti 2p3/2 and Ti 2p1/2 orbitals of metallic Ti show a typical binding energy of 454.2 eV and 460.2 eV, respectively. This peak shift of around 4 eV towards higher binding energy indicates a Ti^4+^ oxidation substituting one Fe^3+^ ion in the hematite lattice, as reported by Wang *et al*.^3^. Therefore, every single Ti^4+^ adds one electron in the hematite films, as shown in Fig. 4(a). The question of different conductivity in different samples remains unexplored because all the samples were doped by 5% Ti and it is highly unlikely that the location of Ti in the hematite lattice changed on annealing, as indicated in Fig. 3(c). In order to have better idea of the films’ composition, the atomic ratio was calculated by using following relation^33^,-

2 $${X}_{i}=100\times \frac{{A}_{i}}{{\sum }_{j=1}^{m}{A}_{j}}$$

Where, X_i_ is the atomic percentage of i^th^ element for a composition of m number of elements. The X_i_ can be determined by adjusted intensity A_i_ of the corresponding element divided by summation of adjusted intensities of all the m number of elements, as indexed by j. Here, the A_i_ can be calculated from the XPS data using following equation –

3 $${A}_{i}=\frac{{I}_{i}}{T({E}_{i})\times {R}_{i}\times {{E}_{i}}^{n}}$$

Where, I_i_ is the peak intensity, T(E_i_) is the transmission function as a function of kinetic energy E_i_, R_i_ is the relative sensitivity factor for i^th^ element, and n is the escape depth. The calculated values of atomic percentage (at. %) are plotted in Fig. 3(d). It was found that Ti at. % in all the samples were almost unchanged with a value of around 4.1%. In an ideal Fe_2_O_3_ films, O-to-Fe ratio should be around 1.5. Here, O/(Ti+Fe) ratio was calculated considering Ti replaces Fe sites in hematite crystal lattice, as evident from Fig. 3(c), denying presence of ionic Ti. The atomic ratio [O/(Ti+Fe)] was found to be below 1.5. This signifies that the films are nonstoichiometric and O deficient. The as-grown sample was observed to have the lowest O/(Ti+Fe) ratio of 1.32 eV, whereas this value for the samples S1, S2, S3, and S4 was calculated to be 1.34, 1.38, 1.42 and 1.45. This oxygen deficiency can be attributed to the presence of either intestinal Fe or oxygen vacancies, or both of them. Because both of them can introduce shallow donor levels in the hematite lattice^34,35^. However, it was reported that iron interstitials have very high activation energy and it can be induced at a very high temperature (≈900 °C)^35,36^. Thus, it was assumed that oxygen vacancies were induced in the hematite films. On the other hand, oxygen vacancies can be either neutral or ionized. According to previous report, ionized oxygen vacancies can contribute to the electrical conductivity by introducing shallow donor levels below the conduction band minimum of hematite^37^. In this context, it was observed that electrical conductivity has a direct relationship with the oxygen deficiencies of the films, as shown in Figs. 2(d) and 3(d). Therefore, it was assumed that ionized oxygen vacancies were induced in the hematite films. Presence of substitutional Ti^+4^ replacing Fe^3+^ and ionized oxygen vacancies gave rise to additional charge carriers in hematite films enhancing its conductivity, as shown in Fig. 4(a),(b), respectively. Upon annealing in oxygen-rich ambient, O atoms slowly diffused to the vacant oxygen sites reducing the conductivity of the films.

**Figure 5:**
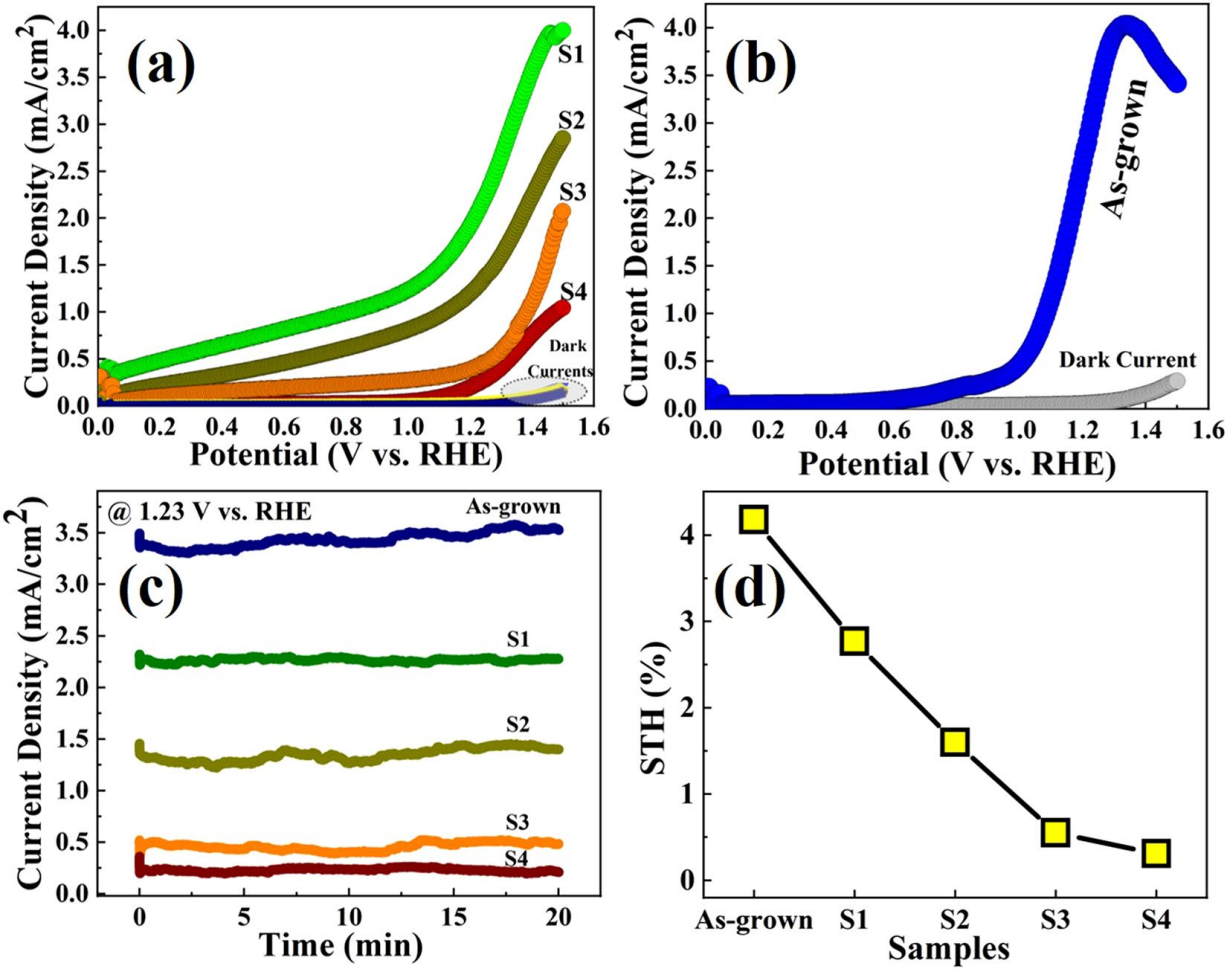
Photoelectrochemical water splitting performance of the hematite samples. (**a**) Dark and photocurrent densities of samples S1, S2, S3, and S4 as a function of voltage vs. RHE. Sample with relatively higher conductivity shows a higher photocurrent. (**b**) Dark and photocurrent densities of as-grown sample. The kink in photocurrent at around 1.35 V vs. RHE was attributed to hindering of reaction kinetics by surface states and functional groups. (**c)** Photocurrent densities of all the samples (as-grown + S1 + S2 + S3 + S4) as a function of time at 1.23 V vs. RHE indicating high quality films and its stability in aqueous environment. (**d**) Solar-to hydrogen conversion efficiencies (STH) calculated at 1.23 V vs. RHE for all the samples.

**Figure 6:**
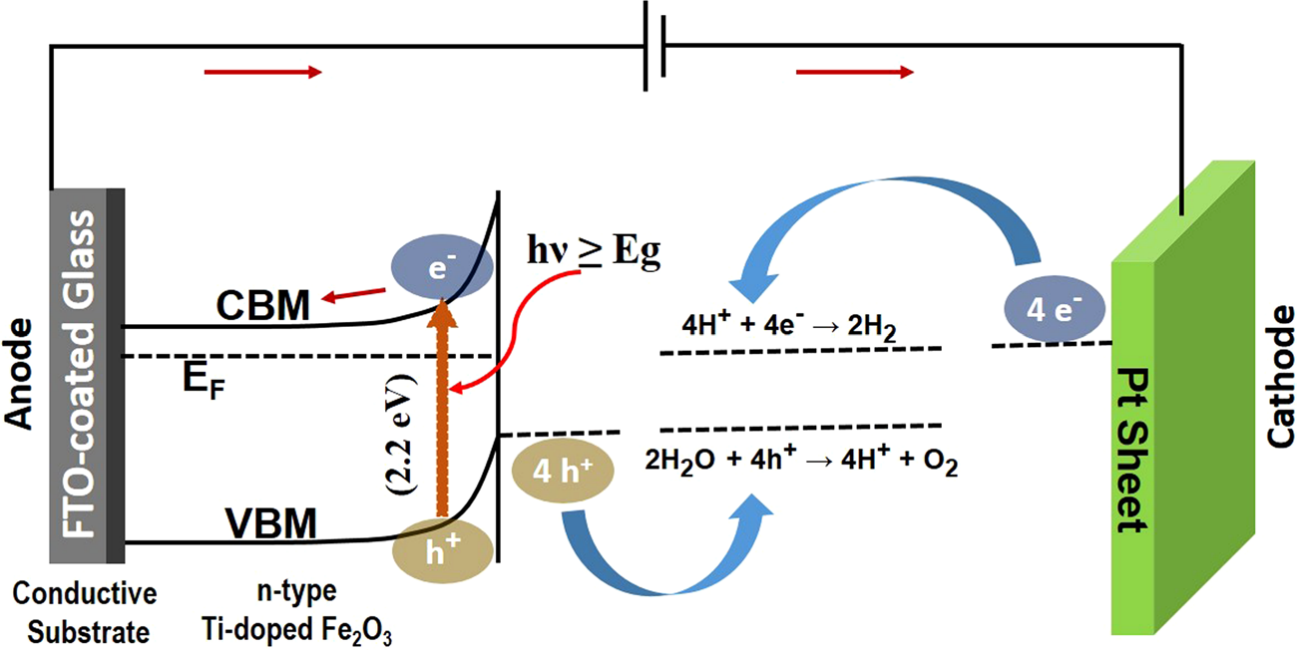
Scheme illustrating a simplistic photoelectrochemical cell comprising n-type Ti-doped hematite films deposited on FTO-coated glass, which acts as photoanode, an external circuit for charge transport and a Pt sheet as the cathode. On exposure to photons with an energy of hv ≥ E_g_, OER initiates on hematite surface while HER starts on Pt sheet.

Figure 5 shows the photocatalytic performance of hematite film which act as a photoanode. The oxygen evolution reaction (OER) (2H_2_O + 4 h^+^ → 4 H^+^ + O_2_) is conducted on hematite and produces oxygen, whereas the hydrogen evolution reaction (HER) (4 H^+^ + 4e^−^ → 2H_2_) takes place as a counterpart of the whole PEC reaction on the cathode (Pt sheet), splitting water molecules in the electrolyte, as shown in Fig. 6. On illumination by sunlight, hematite absorbs the photons with an energy of hv ≥ E_g_, and generates electron-hole pairs. The holes in the valence band initiates OER, whereas the electrons in the valence band transfer to the cathode through the external circuit and facilitate HER on Pt sheet. This reaction cycle does not need an external bias. However, an external bias can accelerate the reaction even at low applied voltage. Figure 5(a) shows the photocurrent and dark current densities as a function of voltage vs. RHE for samples S1-S4. It was observed that samples with relatively lower resistivity and higher carrier concentration have early set-on and higher photocurrent. Amongst all the samples, the photocurrent density for as-grown samples at 1.23 V vs. RHE show a highest value of around 3.4 mA/cm^2^, as shown in Fig. 5(b). However, a kink was observed at around 1.35 V vs. RHE and the photocurrent decreased afterwards. This sudden decrease was attributed to a change in reaction kinetics possibly due to surface states or, functional groups on the as-grown samples. It is believed that these states were eliminated or, minimized in other samples upon annealing. A shift in onset voltage for samples S1-S4 was observed, as shown in Fig. 5(a). This was attributed to the shift in Fermi level due to change in carrier concentration. On the other hand, despite the most conducting sample, as-grown film shows a higher onset voltage in comparison to sample S1, as shown in Fig. 5(b). This was attributed to the post-growth surface states and poor crystallinity of the film. These states were considered to be eliminated on annealing. Figure 5(c) represents the photocurrent density at 1.23 V vs. RHE for all the samples as a function of time. The photocurrents were found to be significantly stable even after 20 minutes of continuous reaction at 1.23 V vs. RHE fixed dc bias. The scanning electron microscopic (SEM) images of the samples before and after reactions revealed similar surface morphologies, as shown in Fig. SI 3(a-j) in the supplementary information. These results indicate that the hematite films are stabile in aqueous environment and signifies that the photocorrosion was negligible. The solar-to-hydrogen efficiency was calculated by using following relationship^38,39^,–

4 $$STH( \% )=\frac{{J}_{p}(mA\cdot c{m}^{-2})\,\times \,1.23\,(V)}{W\,(mW\cdot c{m}^{-2})}\times {\eta }_{F}\times 100$$

Where, J_p_ is the photocurrent density; W is the incident light power density; and is the Faradaic efficiency for hydrogen evolution. Considering ${\eta }_{F}\,$value as unity, as-grown sample with maximum oxygen vacancies was found to be the best-performed photoanode with an STH conversion efficiency of 4.18%, whereas this value for S1, S2, S3, and S4 samples were calculated to be 2.77, 1.60, 0.55, and 0.31%, respectively. It is reported that ionized oxygen vacancies in hematite films retards the electron-hole recombination positively contributing to the photocatalytic performance^37^. The efficiency was reduced for samples with lower electrical conductivity establishing a direct relationship between them.

## Summary

In this paper, we have grown high quality and highly conductive n-type Ti-doped hematite films by spray pyrolysis. The electrical conductivity was tuned as a function of post-deposition annealing temperature in oxygen-rich condition and we systematically investigated the origin of that change in conductivity by using spectroscopic measurements. It was found that oxygen vacancies play a major role in determining the carrier concentration apart from Ti doping. The as-grown samples, deposited in oxygen-depleted ambiance, showed a significantly high carrier concentration of 1.09×10^20^ cm^−3^, and a lower resistivity of 5.9×10^−2^ Ω-cm, which was attributed to the induced oxygen vacancies. The carrier concentration reduced in the annealed samples. The XPS spectra confirmed the purity of the films and oxidation states of Fe and Ti. Significant amount of ionized V_O_ was induced in as-grown samples, which was eventually decreased upon annealing in O-rich conditions. The PEC measurements revealed a direct relationship between conductivity and photocurrents. It was also observed that the films are significantly stable in aqueous environment. The highest STH conversion efficiency for as-grown sample was calculated to be 4.18% at 1.23 V vs. RHE. It was concluded that ionized oxygen vacancies were induced in films that facilitated better conductivity as well as better STH efficiency in the samples.

## Methods

### Hematite film deposition

Titanium-doped hematite thin films were deposited on conducting (≈10 Ω) FTO (fluorine-doped tin oxide)-coated glass substrates by spray pyrolysis. The growth processes were conducted in an oxygen-poor conditions in a closed chamber with positive pressure of N_2_ gas. In order to grow Ti-doped hematite thin films, iron (III) acetylacetonate (≥97%) was used as the Fe precursor, whereas titanium diisopropoxide bis(acetyleacetonate) with a density of 0.995 g/ml in isopropanol was used as the Ti precursor. All the iron oxide films were doped by 5-mol% of Ti. Here, spray solution was prepared by dissolving 0.88 g (50 mMol) of iron (III) acetylacetonate and 45.5 mg (2.5 mMol) of titanium diisopropoxide bis(acetyleacetonate) in 50 ml of (>99.8%) pure methanol. The Fe and Ti precursors were supplied by Sigma-Aldrich and methanol was supplied by Fisher Scientific.

All the growths were performed for 15 min with a flow rate of 2.5 ml/min in an oxygen-depleted condition at a substrate temperature of 450 °C. Nitrogen was used as the carrier gas. Before spraying the solution, nitrogen ambiance was created in the chamber and the substrate temperature was raised to 450 °C. After deposition, the samples were annealed in oxygen-rich condition at 100, 200, 300, and 400 °C and denoted as S1, S2, S3, and S4, respectively, as summarized in the Table 1.

### Materials characterization

The thickness of the samples was measured to be around 180 nm by using a Dektak 6 M Stylus Profiler. Perkin Elmer UV-Vis spectrophotometer lambda 650 was used to study the optical properties of the films. In order to investigate the chemical properties of the films, Omicron multiprobe X-ray photoelectron spectroscopy (XPS) coupled with monochromatic Al Kα X-rays (hv = 1486.7 eV) and an EA125 analyser were used. All the films were sputtered for 15 min before XPS scanning. The crystallographic properties of the films were studied by X-ray diffraction (XRD) by using Bruker D8 Discover coupled with a monochromatic Cu Kα X-ray source. Seebeck coefficients and sheet resistance of the samples were measured as a function of temperature in a vacuum chamber using Keithley measurement units. Sheet resistance measurements were performed with a linear probe configuration. Zeiss ULTRA plus high-resolution field emission scanning electron microscope (FESEM) was used to study surface morphology of the films before and after photoelectrochemical measurements.

### Photocatalytic measurements

Redoxme MM PEC 15 ml double-sided - magnetic mount photo-electrochemical cell equipped with 30 mm Ag/AgCl reference electrode, 50HX15 0.6/250 MM platinum wire counter electrode, and EmStat USB powered potentiostat was used to carry out the photocatalytic performance of hematite thin films as anode. In order to illuminate the photoanode, solar simulator equipped with xenon lamp was used at AM1.5 with an intensity of 100 mW•cm^−2^. Here, Merck supplied 1.0 M phosphate buffer solution with a pH of 7.4 at 25 °C was used as the electrolyte solution. The dc bias voltage (vs Ag/AgCl) was converted into the scale of reversible rydrogen electrode (RHE) by using the relationship:

$${\rm{V}}\,{\rm{vs}}.\,{\rm{RHE}}=({\rm{V}}\,{\rm{vs}}.\,{\rm{Ag}}/{\rm{AgCl}})+0.198+0.06\times {\rm{pH}}=({\rm{V}}\,{\rm{vs}}.\,{\rm{Ag}}/{\rm{AgCl}})+0.642$$

The active area of the samples that was illuminated and exposed to the electrolyte has an area of 1 cm^2^. Figure 6 shows a schematic diagram of the experimental setup essentially representing a photoelectrochemical cell including n-type Ti-doped α-Fe_2_O_3_ as the photoanode and platinum (Pt) sheet as the cathode.

## Supplementary information

Supplementary information.
