# Supplementary material for: Tuning of oxygen vacancy-induced electrical conductivity in Ti-doped hematite films and its impact on photoelectrochemical water splitting
Source: Sci Rep. 2020 May 4;10:7463. doi: 10.1038/s41598-020-64231-w (PMC7198511; doi:10.1038/s41598-020-64231-w)
Supplement: Supplementary file 1 — Supplementary information. [file 41598_2020_64231_MOESM1_ESM.pdf]

Supplementary Information

Tuning of oxygen vacancy-induced electrical conductivity in Ti-doped hematite films and its impact on photoelectrochemical water splitting

Pranab Biswas[1], Ardak Ainabayev[1], Ainur Zhussupbekova[1], Feljin Jose[2], Robert O'Connor[2], Aitkazy Kaisha[1], Brian Walls[1], & Igor V. Shvets[1]*

[1]School of Physics and Centre for Research on Adaptive Nanostructures and Nanodevices, Trinity College Dublin, Dublin 2, Ireland

[2]School of Physical Sciences, Dublin City University, Glasnevin, Dublin 9, Ireland

*Correspondence email: biswasp@tcd.ie

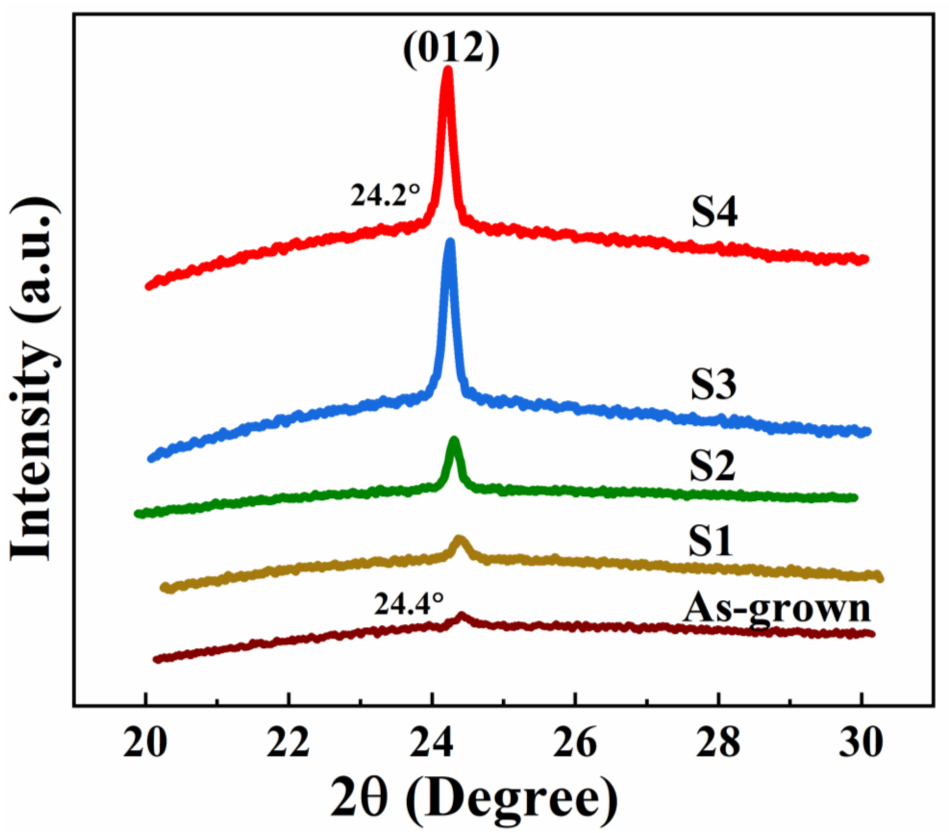

Figure SI 1: Overlay of (012) XRD peaks of the as-grown, S1, S2, S3, and S4 samples indicating the shift in peak on annealing.

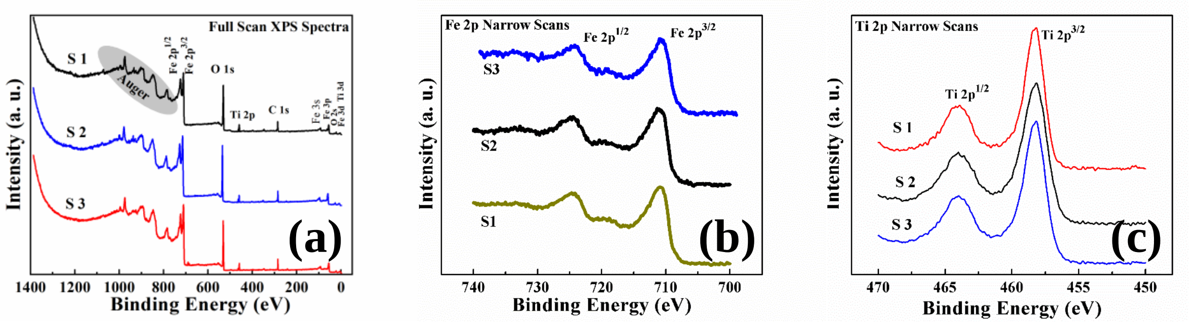

Figure SI 2: (a) Full scan XPS spectra of samples S1, S2, and S3 indicating the composition of the spray deposited films. (b) Fe 2p narrow scan XPS spectra of samples S1, S2, and S3 indicating the oxidation state of Fe in the samples. (c) Ti 2p narrow scan XPS spectra of samples S1, S2, and S3 revealing the oxidation state and the location of Ti in the hematite films.

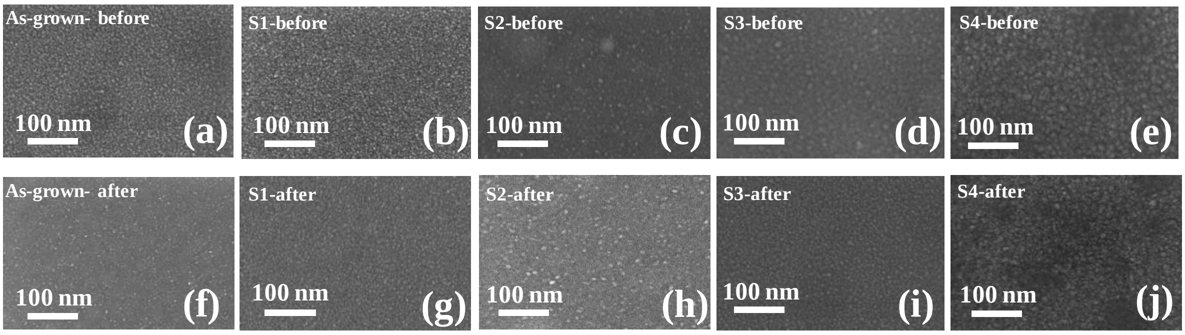

Figure SI 3: Scanning electron microscopic (SEM) images of as-grown, S1, S2, S3, and S4 samples (a-e) before operation and (f-j) after operation, respectively. The images indicate significant stability of the films and negligible photocorrosion occurred during reactions.
